# Supplementary material for: The interactome of intact mitochondria by cross-linking mass spectrometry provides evidence for coexisting respiratory supercomplexes
Source: Mol Cell Proteomics. 2017 Dec 8;17(2):216–32. doi: 10.1074/mcp.RA117.000470 (PMC5795388; doi:10.1074/mcp.RA117.000470)
Supplement: Supplemental Data [file supp_RA117.000470_133922_0_supp_23978_fzffwf.zip › spectra_annotation/mito_spectra_annotation/413-1-1-1-12-1.pdf]

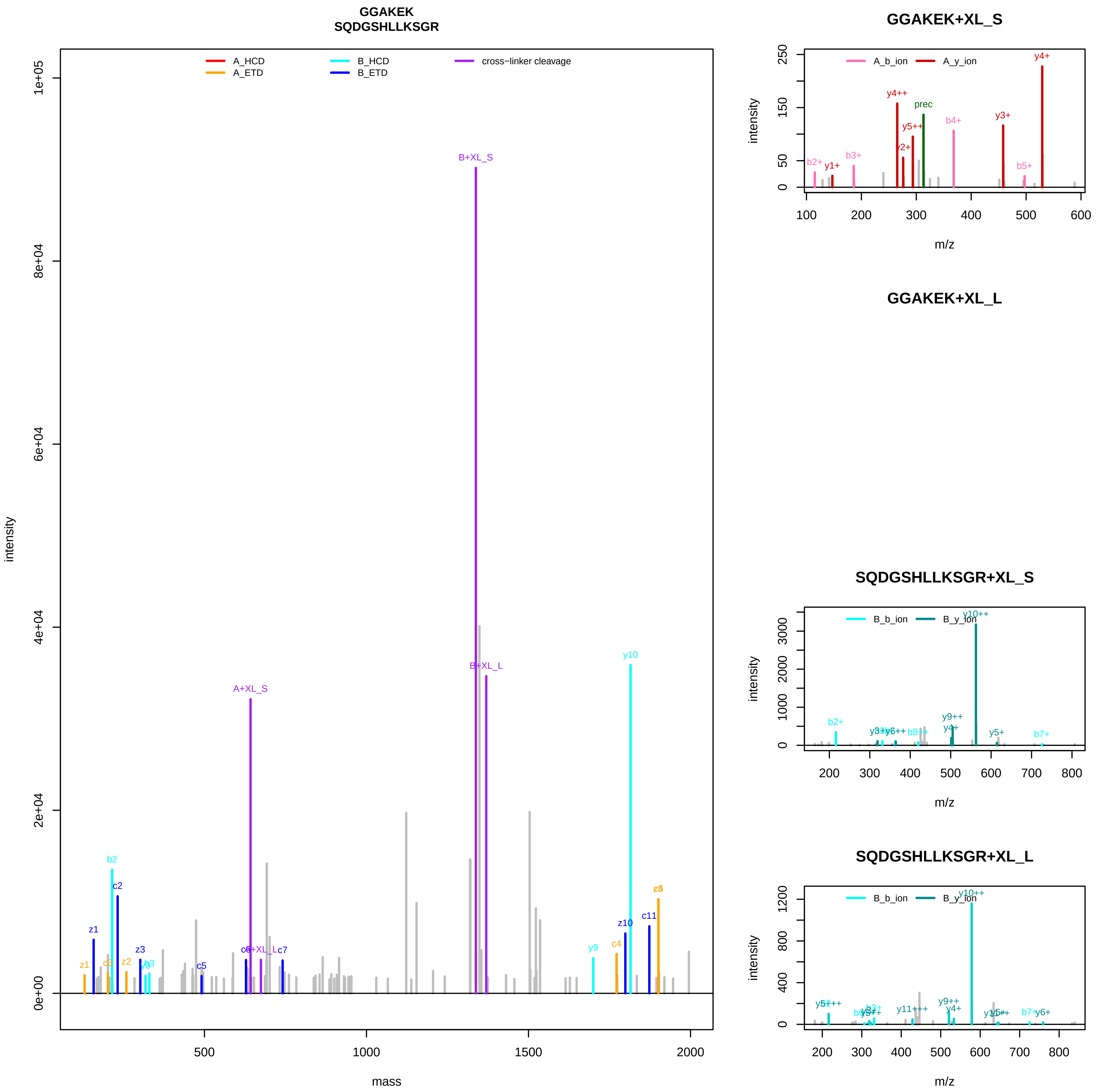
GGAKEK
SQDGSHLLKSGR
A_HCD
A_ETD
B_HCD
B_ETD
cross−linker cleavage
B+XL_S
B+XL_L
A+XL_S
y10
b2
c2
z1
z3
c5
c6+XL_L
c7
y9
c4
z10
c11
z5
z1
c3
z2
y3
b3
intensity
mass
0e+00
2e+04
4e+04
6e+04
8e+04
1e+05
500
1000
1500
2000
GGAKEK+XL_S
A_b_ion
A_y_ion
y4+
y4++
prec
y5++
b4+
y3+
y2+
b2+
y1+
b3+
b5+
intensity
m/z
GGAKEK+XL_L
SQDGSHLLKSGR+XL_S
B_b_ion
B_y_ion
y10++
b2+
y9++
y4+
y6++
b8++
y5+
b7+
intensity
m/z
SQDGSHLLKSGR+XL_L
B_b_ion
B_y_ion
y10++
y9++
y4+
y11+++
b7+
y6+
intensity
m/z
